# Supplementary material for: Competitive Sperm-Marked Beetles for Monitoring Approaches in Genetic Biocontrol and Studies in Reproductive Biology
Source: Int J Mol Sci. 2022 Oct 20;23(20):12594. doi: 10.3390/ijms232012594 (PMC9604355; doi:10.3390/ijms232012594)
Supplement: Supplementary file 1 [file ijms-23-12594-s001.zip › ijms-1920316-supplementary.pdf]

International Journal of
Molecular Sciences

MDPI

Article

Competitive Sperm-Marked Beetles for Monitoring Approaches in Genetic Biocontrol and Studies in Reproductive Biology

Musa Dan'azumi Isah [1,2,†], Bibi Atika [1], Stefan Dippel [1,‡], Hassan M. M. Ahmed [1] and Ernst A. Wimmer [1,*]

1 Department of Developmental Biology, Johann-Friedrich-Blumenbach-Institute for Zoology and Anthropology, Ernst-Caspari-Haus, GZMB, Georg-August-University Goettingen, Justus-von-Liebig-Weg 11, 37077 Goettingen, Germany
2 Department of Crop science, Faculty of Agriculture, Wildlife and Forestry Resources Management, University of Calabar, Calabar P.M. B. 1152, Cross River State, Nigeria
* Correspondence: ewimmer@gwdg.de; Tel.: +49-551-39-28666; Fax: +49-551-39-25416
† Present address: Department of Entomology, 329A Minnie Belle Heep Center, 370 Olsen Blvd, Texas A and M, College Station, TX 77843, USA
‡ Present address: Institute for General Zoology and Developmental Biology, AG Zoology with Emphasis on Molecular Developmental Biology of Animals, Justus-Liebig-University Giessen, Carl-Vogt-Haus R106, Heinrich-Buff-Ring 38, 35392 Giessen, Germany

Supplementary Materials:

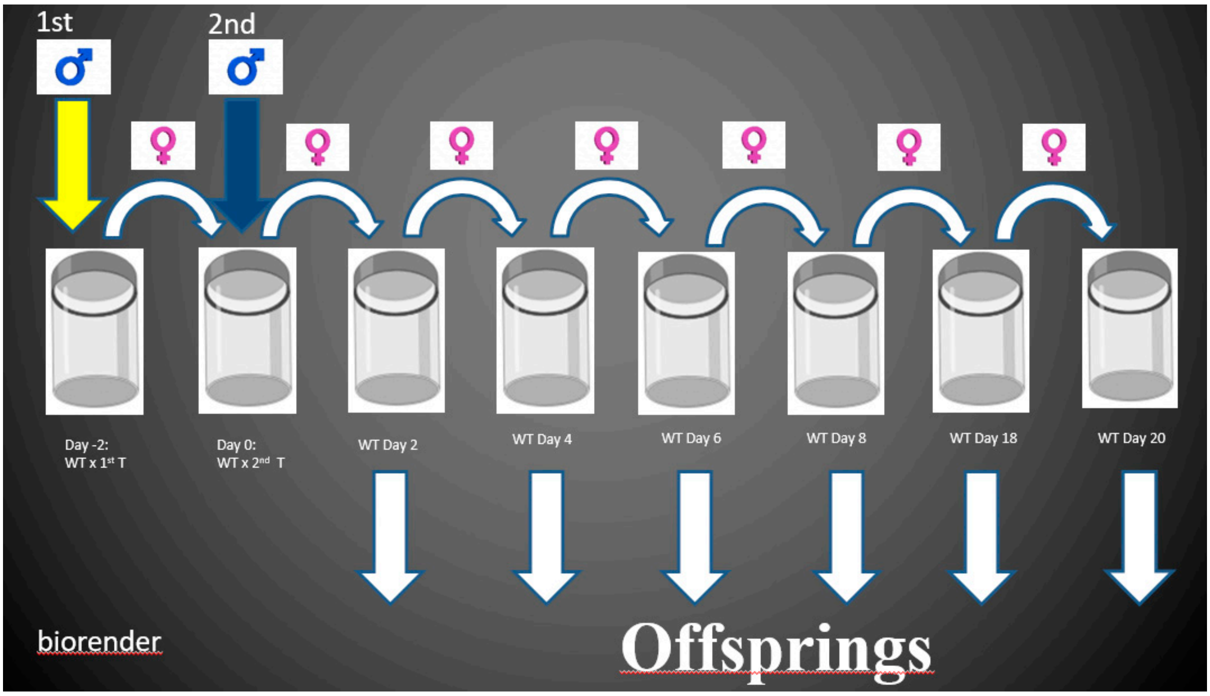

Supplementary Figure S1: Experimental protocol for Sperm utilization and competition of sequentially twice mated females.

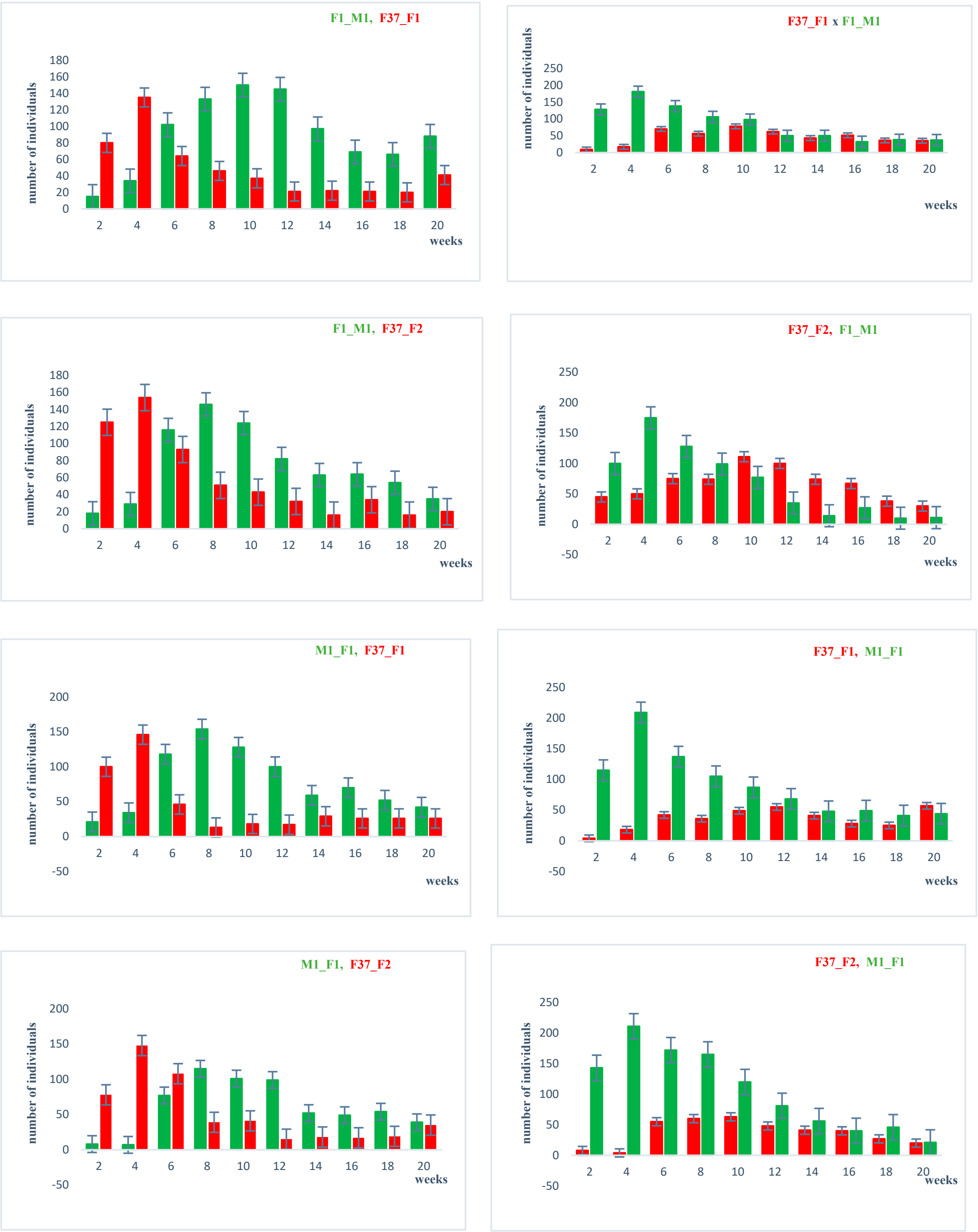

Supplementary Figure S2: Sperm utilization and competition of sequentially twice mated females. Matings were performed as indicted in Supplementary Figure S1 with the order of the respective lines as indicated above each diagram.

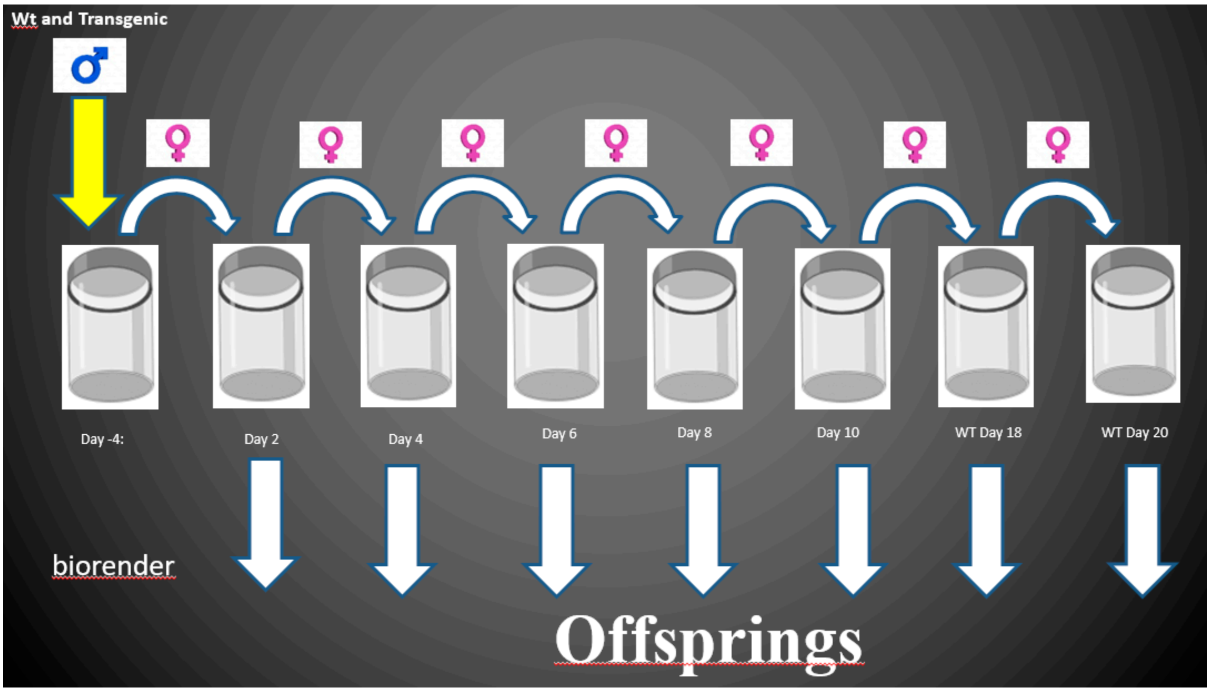

Supplementary Figure S3: Experimental protocol for simultaneous competition in a relaxed (5 transgenic males x 5 non-transgenic males x 10 non-transgenic females), a normal (5 transgenic males x 5 non-transgenic males x 5 non-transgenic females), and a high competition (10 transgenic males x 10 non-transgenic males x 5 non-transgenic females).

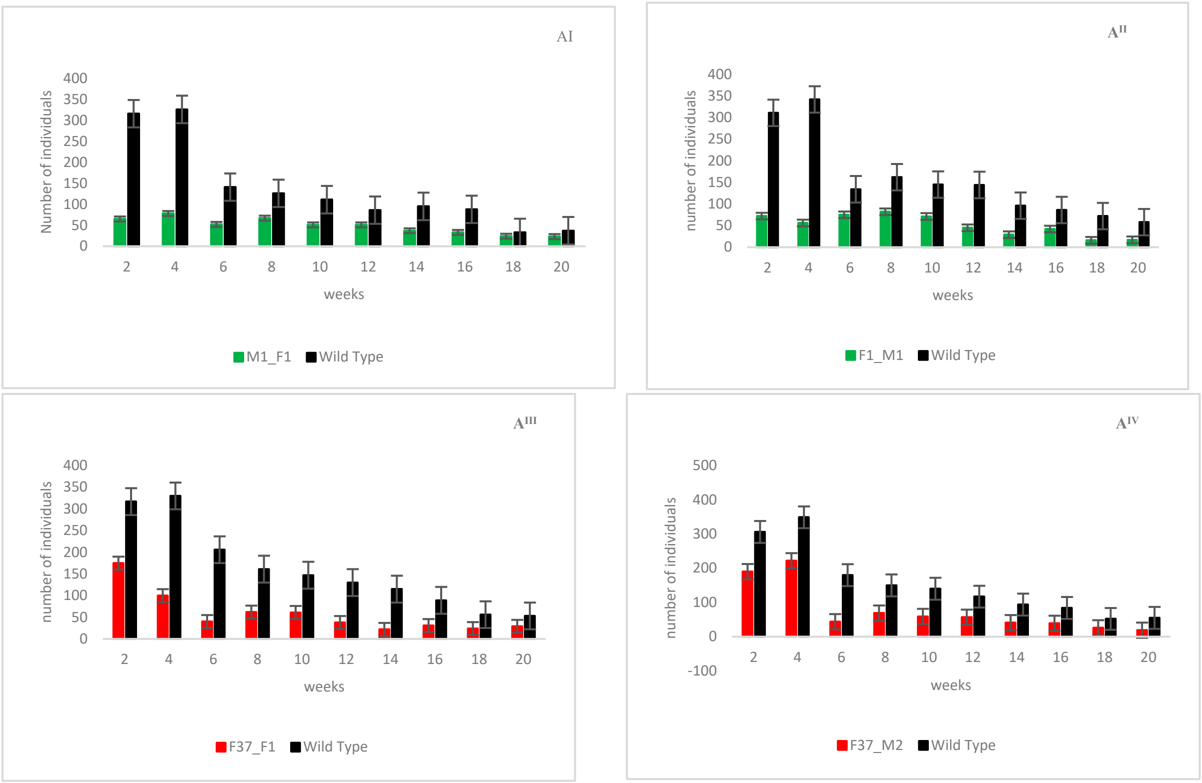

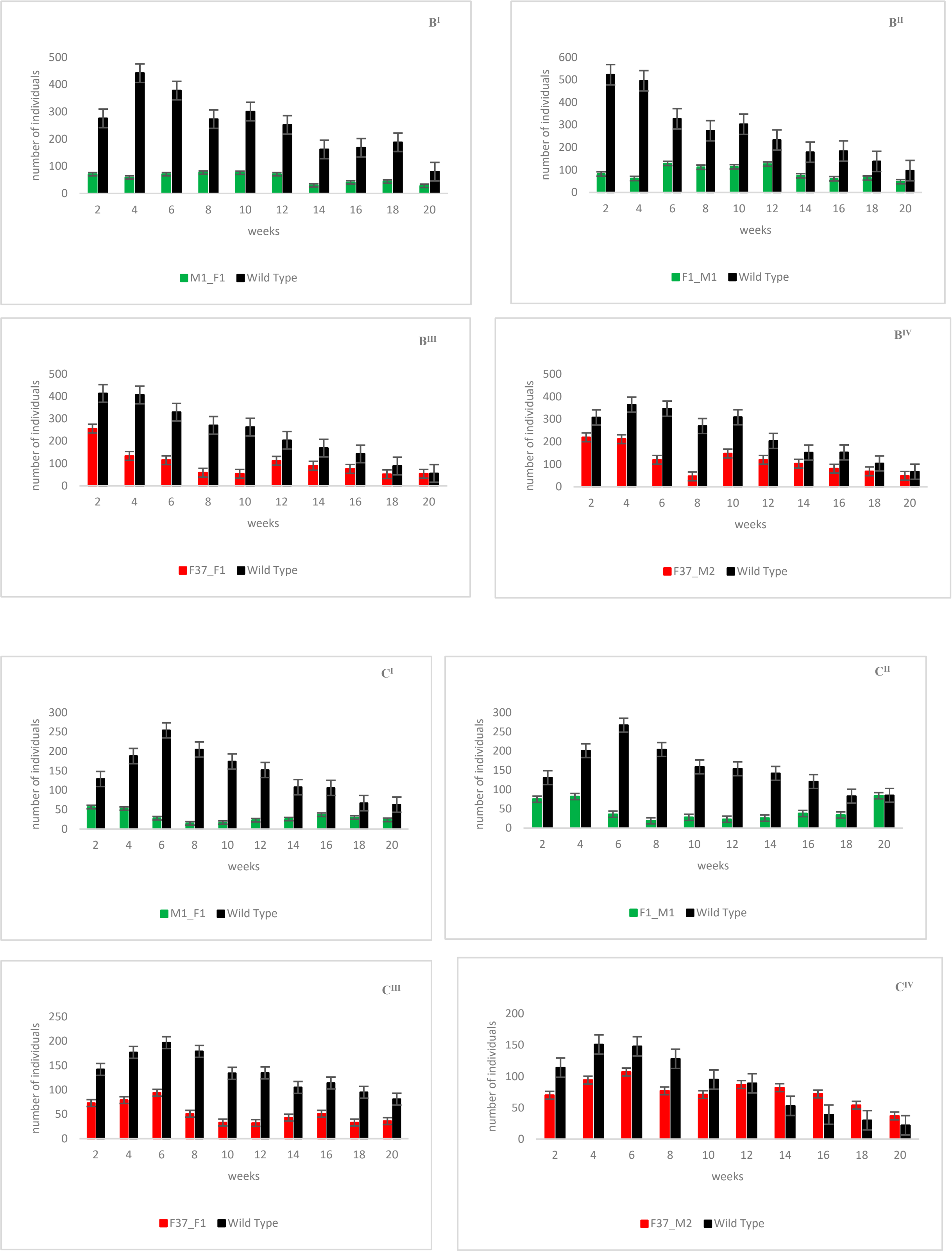

Supplementary Figure S4. Simultaneous competition of transgenic males compared to non-transgenic males. (a): 5 transgenic males x 5 non-transgenic males x 5 non-transgenic females (normal competition). (b) 5 transgenic males x 5 non-transgenic males x 10 non-transgenic females (relaxed competition). (c) 10 transgenic males x 10 non-transgenic males x 5 non-transgenic females (high competition). Matings were performed as indicted in Supplementary Figure S3 with the respective transgenic males competing against non-transgenic males as indicated below of each chart.

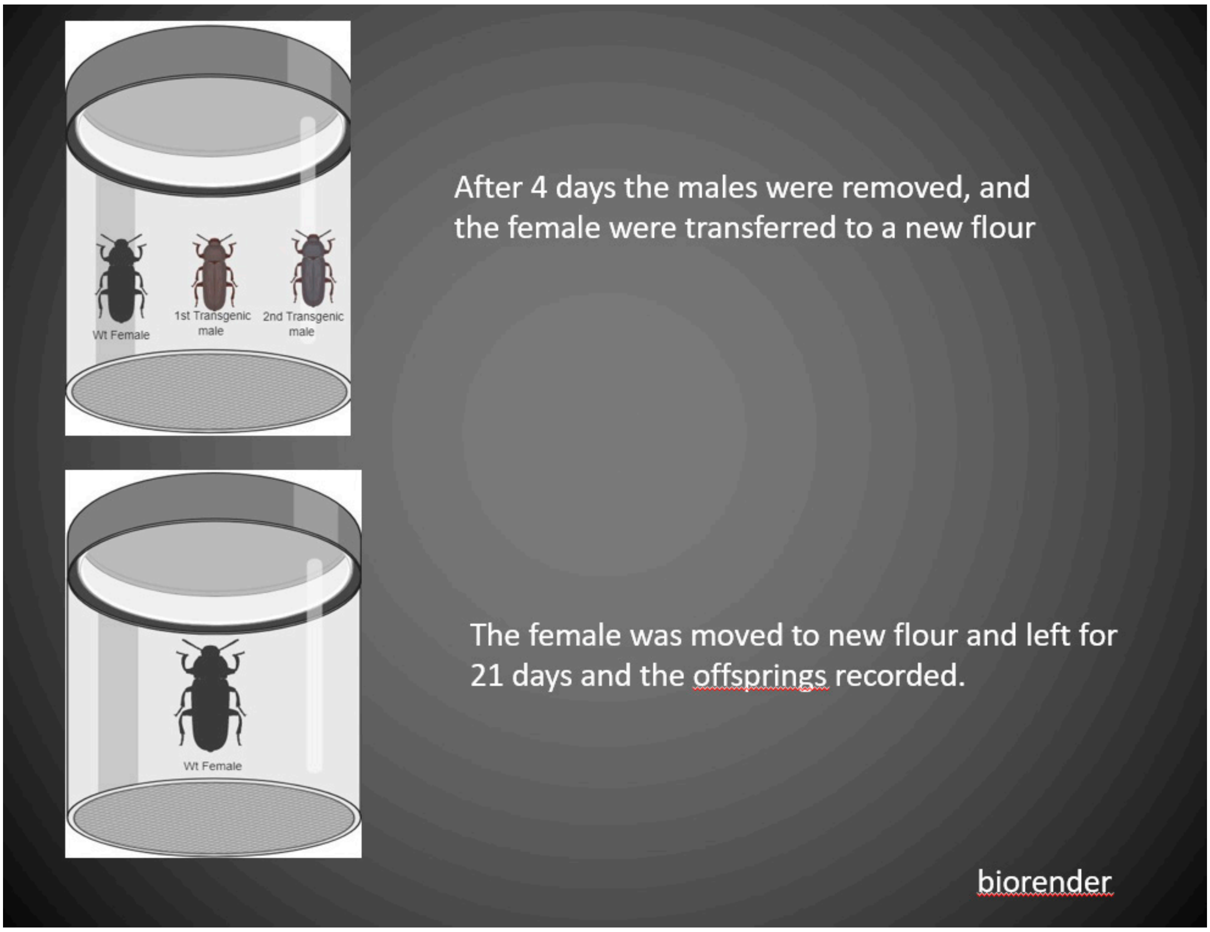

Supplementary Figure S5: Experimental protocol for simultaneous competition between different transgenic males. Ten virgin non-transgenic females crossed with five males of two different transgenic lines each.

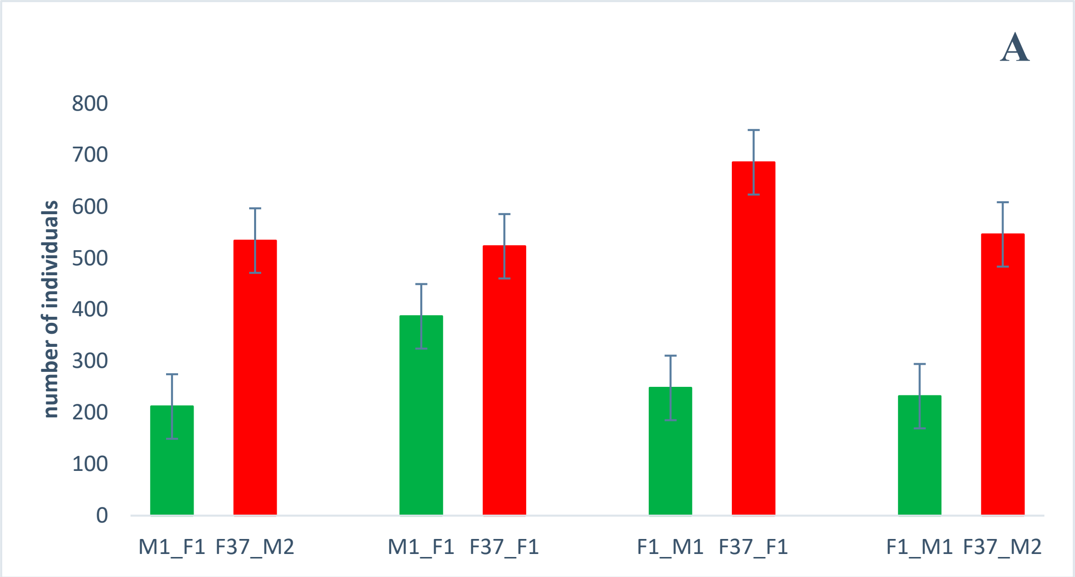

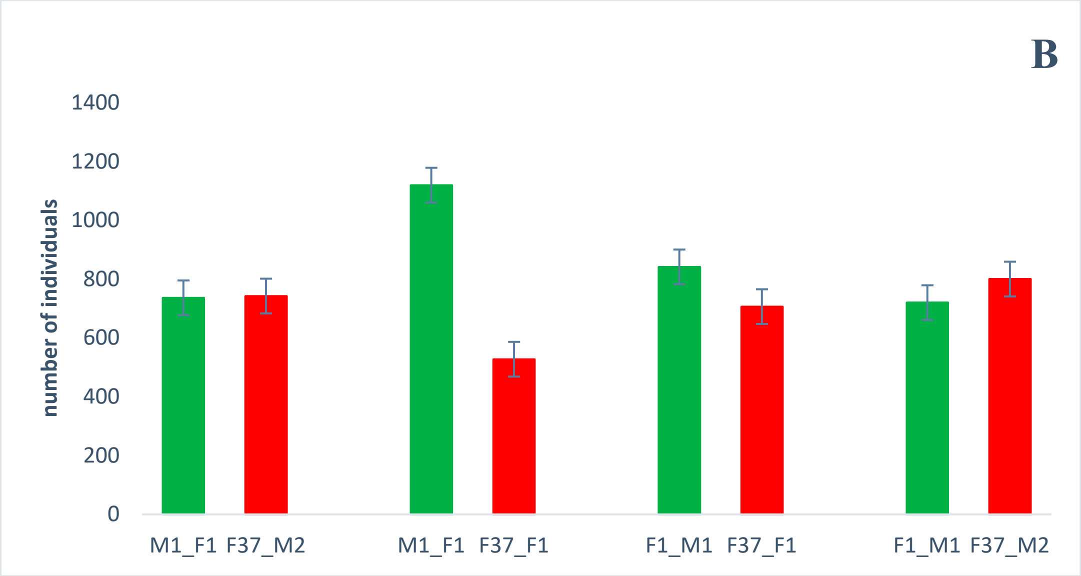

Supplementary Figure S6: Simultaneous competition of different transgenic males. (a) Progeny of the first 4 days after mating. (b) Progeny over 21 days after removal of the males. Matings were performed as indicted in Supplementary Figure S5 with the respective transgenic males competing against each other as indicated below of each column pair.

Supplementary Table S1: Transformation rate

Construct	No. of injected embryos	Hatched larvae	Pupae (G_0)	Crosses with progeny	G_0 with transgenic progeny	Transformation rate
ß2t-egfp	1000	139	77	55	3	5.5 %
ß2t-dsred	1000	210	107	82	4	4.9 %

Supplementary Table S2: List of transgenic lines generated

CONSTRUCT INJECTED	G_0	Transgenic line obtained from G_0
pBac{3xP3_DsRed_SV40af_*attP*-β2t_EGFP_SV40af}	1) F37	F37_F1 and F37_M2
	2) M17	M17_M1
	3) M7	M7_M1
pBac{3xP3_EGFP_SV40af _*attP*_β2t_DsRed_SV40af}	1) M1	M1_M1, M1_M2, M1_F1, M1_F2.
	2) M2	M2_M4, M2_F1, M2_F3
	3) F1	F1_M1, F1_F4, F1_F5, M4_M1, M4_F1, M4_M3
	4) F11	F11_F1, F11_F2, F11_M1, M5_F1, M5_M1

Supplementary Table S3: Integration sites

F1_M1 line = pBac {3xp3_EGFP _SV40-attP_B2t (1Kb) _DsRed_SV40a}

TGCAAAAAAAATACATTGGCTAACAAAAGCAAACGATTTTACTATAATTTTTACTCTTTGTTTCACATAAAATATAAATTCTCTCTAACTTTTACTATAAAAGCATTTAC
TTAACTTTATTCATACGGCTCATTATTTCTATGAACAGTTAATTCCTGCTTTGAAATGCTATGATTATATCCGGTAGGCATTTTAATATTACGTCACCTTAATCACTTT
AATGATTTATTTGTCTTATTTTTTGTGTTAACAATAATTAAATAAATGCATTTTGTTTTTGTTATAATTAGGATTAGAACAATTATTATTTTATTTTAAACAATTTAAA
CAAGT ***TTAA-5'PiggyBac3'-***
TTAATTAATATTAAAAAAATGTTGTTTTTTCCAGCGATTCAATTAAGGGATTAATTATAAAAACAGTATGCTATGTACTCGTATTTAGGAGATAAACTCTGGATCCGGT
GCTCGTAATGATAACAAAAACGGATATTAAATTTCTGATAAGCAGCAATAAAACCTAAT

Integrated in first intron of TC007038.

M1_F1 line = pBac {3xp3_EGFP _SV40-attP_B2t (1Kb)_DsRed_SV40}

TGGTAAATAAGTCCAACATTTTTGAAATAATTTTTATTAGAAAAAAAAGCACAAAAAGTTAATCTTTGCACATCAATCACGATCCTTAATTCTTGTAATAAATTGTTTC
ACCAAAGAAAATAAAGTTGTTCATCCGCAAGCATGATACCACAAAAACAAGACCTCACTTTCCCAGAATTTACAAAATATCCGCGGCATGTCATCCACAATCGCGGCAA
TTTAATTTCCATTTTTATCTTTTCACCGAAAGTAAATCCACTTCCTTGCACGCAAGAAAAATTGTCATTGTGACAATTTATCTCACTATCATCTCTGGTATTGTTTAAT
TAAAAATTTCTCGTCGTTTTTTCACTTTCAACGACGATGATGAGTTCATTATGGAGGCCTTGGCACGTGTTTTCAAATTCGTACGCTTGGCTAG ***TTAA-***
5'PiggyBac3' –
TTAATAAGCTAAATCAGGACCAAAACATGGCAAATATAGTCAATGTAAACACGGGCTGCTGTCCTGTCCATAATCAATATGCGTAAGATGGGTGTTCCGGTTTGGACAC
CTCCTTTCCGGTTTTTAATTCCCATTGCGGCAACGACCTTAGCGCGCCCAGTTTCCGGACACAATAACGTTGGCAGACCTGCCACCATTTTACCAACTTTCT

Integrated in first intron of TC012012.

F37_F1 line = pBac {3xp3_ DsRed _SV40-attP_B2t (1Kb)_ EGFP _SV40}

TGAATTCTTCAATAAATAAATCGTTTAATTGGCCTATCTTGATTAACTGTCATCTTGAAGTTTCAGCTACATACTTTGATTACTAGTTTGAA**TTAA-**
5'PiggyBac3'-TTAA

Integrated in coding sequence of TC032496 (*pangolin*).

Supplementary Table S4: List of primers

ID	NAME	Sequence 5' – 3'
MID#4	Tc-b2t-NcoI 1KB-F	ATGGCCATGGAGGTATCTCACAACCACCGCTTCC
MID#7	Tc- b2t-ClaI-R	GAGGATCGATTTTACTTTAACGTTAAACAAATTTATTAAAGAAATACCG
MID#103	ClaI-EGFP-SV40_F2	GAGGATCGATATGGTGAGCAAGGGCGAG
MID#104	HindIII-EGFP-SV40_R	TTTAAGCTTGATGAGTTTGGACAAACCACAAC
No.5	iPCR5'F1	GACGCATGATTATCTTTTACGTGAC 1st round PCR
No.6	iPCR5'R1	TGACACTTACCGCATTGACA
No:7	iPCR5'F2	GCGATGACGAGCTTGTTGGTG 2nd round PCR
No.8	iPCR5'R2	TCCAAGCGGCGACTGAGATG
No.9	iPCR5'Seq	CGCGCTATTTAGAAAGAGAGAG
MFS227	iPCR3'F1	AGTCAGTCAGAAACAACTTTGGCACATATC
MFS228	iPCR3'R1	CCTCGATATACAGACCGATAAAACACATGC
	3' Junction CH_PRSeq	TACGCATGATTATCTTTAACG
	pJET 1.2 Forward	CGACTCACTATAGGGAGAGCGGC
	pJET 1.2 Reverse	AAGAACATCGATTTTCCATGGCAG
	NcoI-attP-Oligo-F	TCTCCATGGGTGCCCCAACTGGGGTAACCTTTGAGTTCTCTCAGTTGGGGGCGTAGGGTC
	EcoRI-attP-Oligo-R	CTGGAATTCGACCCTACGCCCCCAACTGAGAGAACTCAAAGGTTACCCCAGTTGGGGCAC
